# Efficacy of School-Based Interventions for Improving Muscular Fitness Outcomes in Adolescent Boys: A Systematic Review and Meta-analysis

**DOI:** 10.1007/s40279-019-01215-5

**Published:** 2019-11-15

**Authors:** Ashley Cox, Stuart J. Fairclough, Maria-Christina Kosteli, Robert J. Noonan

**Affiliations:** 1 Movement Behaviours, Health and Wellbeing Research Group, Department of Sport and Physical Activity, Edge Hill University, Ormskirk, UK; 2 Appetite and Obesity Research Group, Department of Psychological Sciences, University of Liverpool, Liverpool, UK

## Abstract

**Background:**

It has been reported that boys’ and girls’ physical activity (PA) levels decline throughout adolescence. Boys are at risk of physical inactivity during adolescence; however, in intervention research, they are an under-represented group relative to girls. It is suggested that the school environment may be central to developing interventions that support adolescents in meeting the current PA guidelines. The aim of this systematic review and meta-analysis was to investigate the efficacy of school-based physical activity interventions for improving muscular fitness (MF) in adolescent males.

**Methods:**

This systematic review and meta-analysis followed the preferred reporting systems for meta-analyses guidelines and was registered on PROSPERO (Registration number: CRD42018091023). Eligible studies were published in English within peer-reviewed articles. Searches were conducted in three databases, with an additional grey literature search in Google Scholar. Studies investigating MF outcomes were included.

**Results:**

There were 43 data sets identified across 11 studies, from seven countries. Overall methodological quality of the studies was moderate-to-strong. Interventions targeting MF evidenced a small-to-medium effect (*g* = 0.32, CI 0.17, 0.48, *p* < 0.00). Subgroup analyses of MF delivery method resulted in small-to-medium effects: upper limb MF measures (*g* = 0.28, 95% CI − 0.02, 0.58, *p* = 0.07), lower limb MF measures (*g* = 0.28, 95% CI 0.09, 0.68, *p* = 0.03), combined MF activities (*g* = 0.24, 95% CI − 0.04 to 0.49, *p* = 0.05), plyometric activities (*g* = 0.39, 95% CI 0.09, 0.68, *p* = 0.01), body weight (*g* = 0.27, 95% CI − 0.10, 0.65, *p* = 0.15), and traditional MF methods (*g* = 0.43, 95% CI 0.09, 0.78, *p* = 0.01).

**Conclusions:**

School-based interventions which aimed to increase MF outcomes in adolescent boys demonstrated small-to-moderate effects. Traditional and plyometric methods of resistance training appear to be the most effective form of PA delivery in adolescent males. More quality research is required to assess the impact of MF delivered in the school environment to inform future intervention design.

**Electronic supplementary material:**

The online version of this article (10.1007/s40279-019-01215-5) contains supplementary material, which is available to authorized users.

## Key Points

**Table:** 

MF interventions delivered in a school-based environment demonstrated small-to-moderate effects in adolescent boys.
MF delivered in a traditional manner, such as weight machines and free weights, may have a greater effect on enhancing MF than other forms of MF delivery.
Plyometric forms of MF delivery demonstrated significant homogeneous effects and require further quality research to assess their application in the school environment.

## Introduction

It is recommended that adolescents engage in a minimum of 60 min of moderate-to-vigorous physical activity (MVPA) per day with muscle and bone strengthening exercise (MBSE) to be incorporated three times per week [1–4]. A recent systematic review confirmed the associated health benefits of meeting the recommended MVPA guideline [5]. Furthermore, participating in the recommended 3 days of MBSE per week has also been associated with positive physical and mental health benefits in children and young people [6–10]. Despite this evidence, less than 50% of young people in Europe meet the recommended amount of MVPA suggested by the World Health Organisation (WHO), with this figure declining with age [11]. There is also an international downward temporal trend in muscular fitness among school children, indicating a lack of activities that support the development of muscular fitness [12–15]. Muscular fitness is assessed by measuring performance in tests of muscular strength, power, and muscular endurance [12], and forms part of the MBSE guideline for PA. Lower levels of muscular fitness are associated with the development of non-communicable disease in adolescent populations [16–21]. Moreover, the development of muscular fitness has been correlated with enhanced bone health, enhanced motor skill, and decreased fat mass in adolescents [22–24].

The benefits of MBSE are well established, supported by position stands from leading organisations [25, 26]. Despite the growing body of literature supporting the benefits of MF, it is often the overlooked element of PA guidelines. Recent UK estimates for health care costs associated with muscle weakness, defined by low grip strength according to the Foundation for the National Institutes of Health criteria (men < 26 kg, women < 16 kg), exceed £2.5 billion [27]. Furthermore, the United States reported estimated health care costs associated with muscular weakness at $18.5 billion [28]. Poor muscular fitness is associated with sarcopenia, poor quality of life, loss of functional movement, and increasing the likelihood of contracting a non-communicable disease [29]. The associated health care costs and accompanying pathologies support the need to address the downward trend in muscular fitness currently witnessed in youth.

The school environment has been shown to be effective in the promotion of PA in adolescents [30]. Adolescents are most active during the school day compared to evenings and weekends [31]. Additionally, the school environment provides access to PA independent of background or socioeconomic status [32]. This may expose adolescents to varying forms of PA that they may not have been exposed to outside of school. However, the efficacy of school-based interventions investigating PA in adolescent males is unclear. Much of the existing research and policy to promote PA is directed towards adolescent girls, suggesting that males are at low risk of not meeting the suggested PA levels indicative of good health [33–36]. However, boys are reported to be at greater risk than girls of becoming overweight or obese, compromising short- and long-term health [36–40]. Recent national surveillance data suggest that adult males may be more likely to be overweight when compared to adult females [41, 42]. Additionally, worldwide trends in BMI are increasing year on year, with Asia displaying a period of acceleration [43]. For male adolescents, healthy behaviours catalysed during adolescence are often carried into adulthood, supporting the need to investigate the efficacy of current interventions [44].

It is hypothesised that male adolescents may respond more favourably towards resistance training (RT) as these activities are perceived as masculine [45, 46]. Furthermore, existing evidence supports the role of MF interventions for improving physiological and psychological health [6, 8, 47]. However, research suggests that the development of MF in upper and lower limbs is not homogeneous, and may vary throughout growth and maturation [48–50]. The heterogeneous nature of MF development in adolescent boys may not be accounted for when prescribing RT on a large scale. Understanding how this phenomenon impacts school-based delivery of RT may support future intervention design when attempting to cater for multiple participants. Additionally, appropriate forms of RT delivery may engage overweight or obese adolescents [51]. Implementing effective RT interventions in the school environment may allow overweight and obese youth to excel by taking advantage of their relatively greater absolute strength [51]. Therefore, RT may be a way of increasing PA levels and improving health among overweight or obese adolescents. However, RT is often an overlooked element of PA guidelines when considering the development of school-based interventions and requires contextualisation.

When exploring the existing literature that reports on the efficacy of MVPA interventions across both sexes and age ranges, mixed outcomes have been reported with small changes of around 4 min per day following school-based interventions [52]. However, it is unclear how adolescent boys respond to school-based RT interventions. To the authors’ knowledge, this review is the first to investigate the efficacy of school-based PA interventions to improve MF outcomes in adolescent boys. This systematic review and meta-analysis will include studies that (1) represent adolescent boys and report MF outcomes; and (2) determine the efficacy of RT interventions delivered in school settings. Thus, the purpose of this systematic review and meta-analysis is to investigate the efficacy of school-based interventions on MF outcomes in adolescent boys.

## Methods

### Protocol and Registration

This systematic review and meta-analysis were registered with PROSPERO on 15th March, 2018 (Registration number: CRD42018091023). The protocol is published online (https://www.crd.york.ac.uk/prospero/display_record.php?RecordID=91023), and follows the PRISMA statement for reporting systematic reviews and meta-analyses.

### Search Procedure

**Fig. 1:**
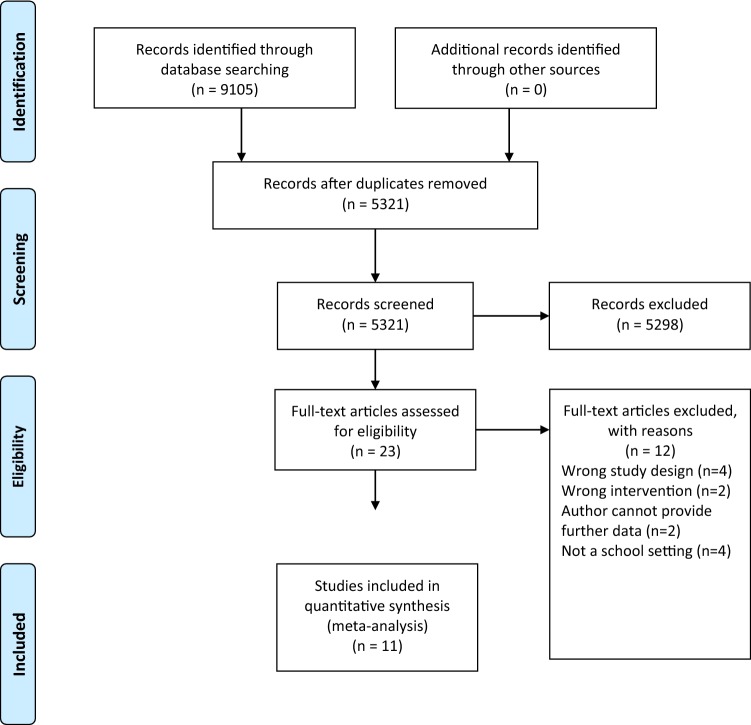
PRISMA flow diagram to show each stage of the systemic eligibility process

A systematic search was conducted in April 2018 using three electronic databases (PubMed, SPORT Discus, and Web of Science). A grey literature search of Google Scholar was also conducted to minimise publication bias [53]. Journal articles published in English post-May 2010 until the date of the final search in August 2018 were considered for review. May 2010 was chosen as the initial reference point to capture all interventions conducted, following the publication of the WHO PA guidelines [1]. WHO guidelines were used as the PA guideline reference to provide a balanced search strategy, accounting for all countries, including those yet to establish their own PA policy and guidelines [54]. The search strategies for each database are detailed in Table S1 as supplementary information, with a link to one of the database searches as per PRISMA guidelines. The PRISMA flow diagram detailing the procedure can be found in Fig. 1. Reference lists of relevant articles, including systematic literature reviews, were examined for potential articles which fitted the criteria. A recent systematic review that reflects the target population group and training intervention for this review was also checked for any further literature [6]. All search results were exported to a reference manager, Covidence (https://www.covidence.org; Covidence, Melbourne, Australia), allowing for central reviewing and collection of all texts for screening.

### Study Selection

Studies were eligible if they contained an intervention where the main purpose was to promote PA in the school environment, with the primary outcome of increasing objectively measured MF. Included studies investigated adolescent boys aged 10–18 years. Mixed boys’ and girls’ data were acceptable if sex-specific results were available and/or accessible. Studies must have been conducted in a school or college between 8 am–6 pm on week days during term time. Studies were included if MF measures were taken at baseline and at the end of the intervention. Girls, community interventions, elite sport, and thesis/dissertations were excluded. Measures of MF had to have been documented in their use previously in peer-reviewed research and could not be novel or first-time iterations of a testing protocol.

Studies could be randomised or non-randomised. Research studies published before 2010 were excluded as were studies that were not published in English. Where full texts were not readily available and where only partial data were reported, the study authors were contacted and asked to provide the full-text version with the accompanying data in full. If no response was received after an 8-week follow-up reminder, these studies were excluded as they could not be fully assessed for eligibility. A total of 11 authors were contacted to provide further data and full texts. From the authors contacted, five non-responses were recorded, with a further two authors unable to provide further data for analysis.

### Data Extraction and Risk of Bias

All search results were exported into Covidence (https://www.covidence.org; Covidence, Melbourne, Australia) and duplicates were removed. The first author (AC) screened all titles and abstracts for obvious irrelevance, 10% were also checked by another author (RN). The 10% screening figure is a recognised validation and agreement threshold for systematic reviews [55]. The full text of eligible studies was then located and reviewed by two authors (AC and RN). Any disagreements were resolved in a meeting involving three authors (AC, RN, and SF). ﻿Study data were extracted by AC and included study characteristics (i.e., country, year); participant characteristics (e.g. sample size, age, anthropometrics); intervention components (i.e., setting, duration, intervention); and changes in the outcomes (i.e., change in grip strength). The outcome data were extracted in the form of mean, standard deviation, and sample size. Included studies were assessed for risk of bias using a modified tool [56, 57] appropriate for PA reviews which included measures for quantitative studies.

### Data Synthesis and Analysis

Random effects meta-analyses were conducted using Comprehensive Meta-analysis Software (Version 2.2.064). Raw scores were converted to standardised means data. Studies that reported more than one measure of assessing a single outcome (i.e., vertical jump height and reactive strength index for lower limb outcome) were converted into a single common effect size for the analysis to avoid inflating sample sizes. A random effects model was considered more appropriate for this review to account for the expected heterogeneity between PA measures [58]. Hedges’ *g* with 95% CIs were used to calculate effect sizes [59]. Pooled weighted standard deviations were used as per the Hedge’s *g* formula and based on a positive effect direction [59]. Hedges’ *g* was interpreted using Cohen’s [59] effect sizes, as small (0.2), medium (0.5), and large (0.8). Heterogeneity was assessed using *I*^2^ statistic, with values of 25, 50, and 75 representing low, medium, and high heterogeneity, respectively [60]. Publication bias was assessed using Egger’s statistic, where bias was deemed to be present at *p* < 0.05 [61]. Corresponding funnel plots were created for visual interpretation, followed by calculating Egger’s statistic to confirm or refute publication bias.

### Quality Appraisal

Included studies were assessed for risk of bias using a modified tool suitable for PA interventions that included non-RCT designs [56, 57]. The ability to distinguish the nature of the PA outcome assessment method in addition to the existent randomisation, blinding, and complete outcome data items was accounted for within this tool. This adapted quality assessment tool used a 1–4 scoring system (i.e., 1 = weak and 4 = very strong; see Table 1.

**Table 1:** Quality assessment (risk of bias)

Study	Appropriate sequence generation and/or randomisation	Allocation concealment and/or blinding	Complete outcome data and/or low withdrawal/dropout (< 20%)	Appropriate outcome measure (PA)	Quality score
1. De Souza et al. (2015) [62]			X	X	2
2. Eather et al. (2016) [63]	X	X	X	X	4
3. Giannaki et al. (2016) [64]		X	X	X	3
4. Kennedy et al. (2018) [65]	X		X	X	3
5. Lloyd et al. (2012) [66]			X	X	2
6. Lloyd et al. (2016) [67]			X	X	2
7. Lubans et al. (2016) [68]	X	X		X	3
8. Muehlbauer et al. (2012) [69]			X	X	2
9. Muntaner-mass and Palou (2017) [70]			X	X	2
10. Weeks and Beck (2012) [71]			X	X	2
11. Winwood and Buckley (2017) [72]			X	X	2

X = the study demonstrated appropriate steps to account for the respective risk of bias confounder

## Results

Extracted studies were conducted in seven countries (UK, Brazil, Australia, Cyprus, Germany, Spain, and New Zealand) [62–72]. The studies included displayed no obvious bias, but rather a lack of depth and detail, which made the risks of bias difficult to detect. Details regarding sequence generation and allocation concealment and/or blinding were found to be the categories that were often not sufficiently reported on. Twenty-seven percent of the studies reported an appropriate sequence generation or randomisation in detail [63, 65, 69], with a further 27% reporting allocation concealment or blinding in detail [63, 69, 72]. This may suggest selection and reporting bias in the literature. Complete outcome data and/or low dropout rates were present in 81% of the included studies and can, therefore, be interpreted as having low risk of bias as a result of attrition. Risk of bias through inappropriate outcome measures was not an issue for this review as all studies selected had to demonstrate an objective way of assessing MF.

**Fig. 2:**
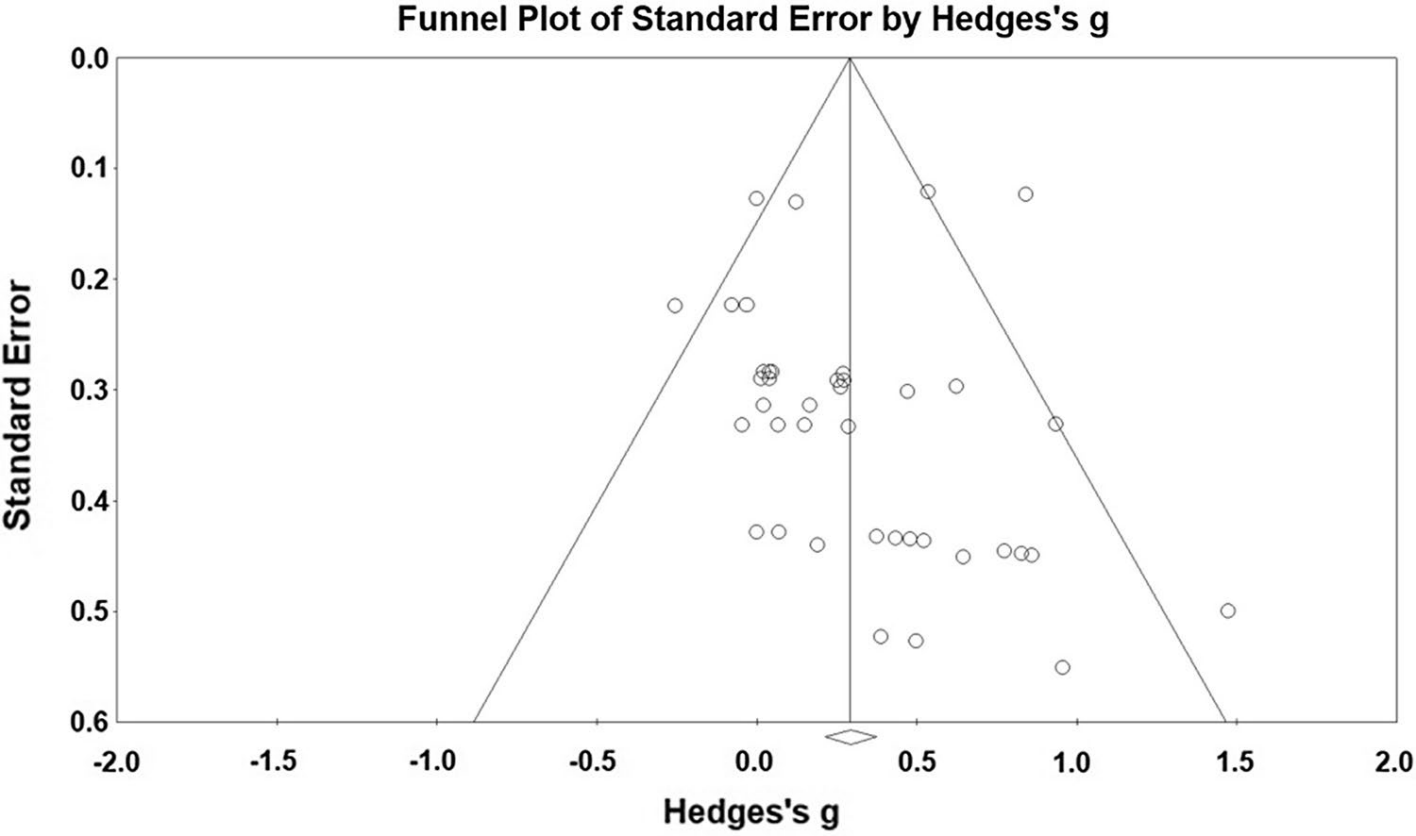
Funnel plot of standard error by Hedge’s g

Forty-three data sets were extracted from 11 studies [62–72] assessing MF, with studies reporting multiple MF outcomes including a combination of upper and lower limb measures. Upper and lower limb data sets were analysed independently to identify possible intervention effects, categorised by testing site. Further subgroup analyses of MF interventions were conducted, accounting for: bodyweight movements (i.e., push-ups and curl ups), combined activities (i.e., the use of multiple forms of resistance exercise such as bodyweight and plyometric within the same intervention), plyometrics, and traditional methods such as weight machines and free weights. Plyometric training studies had to exclusively state that the intervention utilised the stretch-shortening cycle to take advantage of the elastic properties of the muscle to produce power [73, 74]. Participants’ ages ranged from 11.0–16.9 years, samples were separated into, MF control (*n* = 1164) and MF intervention (*n* = 1252). A full breakdown of how sample sizes were extracted is provided in Table 2. Identification of possible publication bias was plotted against standard errors to generate funnel plot (Fig. 2). Egger’s analyses for all data sets suggested that publication bias was not present (*p* > 0.05).

**Table 2:** Characteristics of MF studies included in this systematic review. Letters a–l relate to individual outcome measures and are displayed combined where studies have reported multiple methods for a single outcome in subsequent forest plots

Study and quality rating	Participants	Intervention, duration, and measurement period	PA measurement method and PA outcome measure
De Souza et al. (2015) Brazil [62]	Total: *n* = 19 Mean age: 12.8 ± 0.6 Control: Mass (kg): 54 ± 10 Height (m): 1.57 ± 0.7 BMI: 22 ± 3 Intervention: Mass (kg): 52 ± 9 Height (m): 1.60 ± 0.8 BMI: 20 ± 2	12 weeks, completing two 60 min sessions per week The calisthenics exercise group performed a 10-min warm-up (running) followed by five calisthenics strength exercises: (a) wide-grip push-ups; (b) squat or lunge; (c) fixed bar inverted row; (d) curl ups; and (e) narrow-grip push-ups)	Muscle/bone strengthening (a) Horizontal jump: the subjects performed the horizontal jump test. The best distance (in centimetres) of three attempts was recorded (b) Push-ups in 1 min: the subjects performed the maximum number of repetitions in 1 min (c) Curl ups: the subjects performed the maximum number of repetitions in 1 min
Eather et al. (2016) Australia [63]	Total: *n* = 46 Mean age: 15.3 ± 0.47 Mass (kg): 65.1 ± 12.3 Height (m): 1.77 ± 0.72 BMI: 21.3 ± 3.4 Control: *n* = 22 Intervention: *n* = 24	8 weeks, completing 2, 60 min sessions per week Sessions were delivered by crossfit coaches. A typical session included a dynamic warm-up (10 min), a technique-based skill session (10 min), a workout of the day (10–20 min), a stretching session (5–10 min), and time allocated for organisation, transition, and changing into sportswear (10 min)	Muscle/bone strengthening (a) Push-up tests (reps) (b) Curl up test (reps) (c) Standing jump (m) (d) Grip strength (Kg) Guided by the FitnessGram protocol
Giannaki et al. (2016) Cyprus [64]	Total: *n* = 39 Mean age: 16 Control: *n* = 19 Mass (kg): 59.4 ± 13.7 Height (cm): 169.3 ± 8.9 BMI: 20.5 ± 2.9 Intervention: *n* = 20 Mass (kg): 64.5 ± 13.0 Height (cm): 169.8 ± 6.4 BMI: 22.3 ± 3.7	8 weeks, completing two sessions per week Circuit training was performed in a group setting, where the students completed 20 min consisted of two cycles of eight exercises (stations) with 30-s exercise—30-s rest between sets and 3-min rest between cycles. The circuit training included push-ups, tricep dips, step-on-the-box, wall ball (squats holding a 2 kg medicine ball and then throwing the ball on the wall on the ascent), bicep curls with elastic bands for resistance, sit-ups, standing calf raises with medicine ball, and back raises. The circuit training program was altered in the last 4 weeks of the intervention. Changes were made both in the volume and frequency of the exercises, reaching the total number of exercises (stations) to 10, whilst the resting period between each exercise was reduced by 15 s	Muscle/bone strengthening (a) Hand-grip strength (kg) left (b) Hand-grip strength (kg) right (c) Vertical jump (cm)
Kennedy et al. (2018) Australia [65]	Total: *n* = 303 Control: *n* = 124 Mean age: 14.2 ± 0.5 Intervention: *n* = 179 Mean age: 14.1 ± 0.4	10-week school term, with pre-test and post-test data collection occurring in the preceding and ensuing school terms to the intervention, respectively [i.e., pre-tests occurred in term 2 (April–June), the intervention was delivered in term 3 (July–September), and post-test occurred during term 4 (October–December)]. This resulted in an approximate period of 6 months between pre-test and post-test measurements The structured physical activity program followed a specified session format, including: (i) movement-based games and dynamic stretching warm-up; (ii) RT skill development; (iii) high-intensity RT (HIRT) workout; (iv) modified game involving fitness infusion, boxing or core strength activity; (v) static stretching, reinforcement of behavioural changes	Muscle/bone strengthening (a) Push-ups (reps) (b) Standing long jump (m)
Lloyd et al. (2012) UK [66]	Total: *n* = 109 Control 1: *n* = 22 Mean age: 12.23 ± 0.28 Mass (kg): 47.38 ± 13.91 Height (cm): 151.67 ± 6.93 Intervention 1: *n* = 22 Mean age: 12.29 ± 0.31 Mass (kg): 44.78 ± 9.42 Height (cm): 151.89 ± 7.94 Control 2: *n* = 24 Mean age: 15.29 ± 0.33 Mass (kg): 63.70 ± 11.43 Height (cm): 174.11 ± 9.20 Intervention 2: *n* = 20 Mean age: 15.33 ± 0.27 Mass (kg): 64.96 ± 8.89 Height (cm): 174.35 ± 6.63	4 weeks of 2 × sessions per week Training volume was defined by the number of foot contacts made during each session, starting with 72 contacts in the first session, increasing to 106 contacts in the final two sessions. Plyometric drills lasted approximately 5–10 s, and at least 90 s rest was allowed after each set Plyometric drills included standing vertical and horizontal jumps, lateral jumps, ankle hops, skipping, single-leg hopping, maximal hopping, and low-level drop jumps (20 cm) Measurements taken pre- and post-intervention	Muscle/bone strengthening Reactive Strength Index (millimetres per millisecond). Reactive strength index (RSI) was determined during the maximal hopping test, which involved the participants performing five repeated bilateral maximal vertical hops on the contact mat. The participants were instructed to maximise jump height and minimise ground contact time. The first jump in each trial was discounted, whereas the remaining four hops were averaged for the analysis of RSI (a) Intervention 1, pre-peak height velocity (b) Intervention 2, post-peak height velocity
Lloyd et al. (2016) UK [67]	Total: *n* = 80 (*n* = 40 pre-PHV, *n* = 40 post-PHV) Participants were divided into 4 groups, plyometric training, traditional strength training, combined training and control	Two sessions per week for 6 weeks Within traditional strength training sessions, participants completed three sets of ten repetitions of a barbell back squat, barbell lunge, dumbbell step up, and leg press. To enable the prescription of individualized training intensities, ten repetition maximum (10RM) loads were calculated for participants in the traditional strength training group before the start of the training period. Progressive overload (5%) was implemented following technical competency Plyometric training prescription included a combination of exercises that were geared toward developing both safe jumping and landing mechanics (e.g., drop landings, vertical jumps in place, and single-leg forward hop and stick) and also to stress stretch-shortening cycle activity (e.g., pogo hopping, drop jumps, and multiple horizontal re-bounds). Within each session, participants were exposed to multiple sets of four exercises to enable sufficient repetition to develop motor control programs. (week 1 foot contacts = 74 per session; week 6 foot contacts = 88 per session) The combined training program involved exposure to two traditional strength training exercises (barbell back squat and barbell lunge) and two varied plyometric exercises, each session taken from the plyometric training program Measurements taken pre- and post-intervention	Muscle/bone strengthening Squat jump height (cm) Pre-PHV–Post-PHV Plyometric training: a, d Traditional strength: b, e Combined training: c, f Reactive Strength Index (millimetres per millisecond) Pre-PHV–Post-PHV Plyometric training: g, j Traditional training: h, k Combined training: i, l
Lubans et al. (2016) Australia [68]	Total: *n* = 361 Control: *n* = 180 Mean age: 12.7 ± 0.5 Mass (kg): 53.1 ± 13.4 Height (cm): 160.2 ± 8.4 BMI: 20.5 ± 4.1 Intervention: *n* = 181 Mean age: 12.7 ± 0.5 Mass (kg): 54.0 ± 15 Height (cm): 160.9 ± 9.0 BMI: 20.5 ± 4.1	20-week, 20 × 90-min sessions delivered by teachers during school sport periods in addition to regular PE. Lunch time sessions run by students, 6 × 20-min sessions Each session included the following structure: (i) warm-up: movement-based games and dynamic stretches; (ii) resistance training skill development: resistance band and body weight exercise circuit; (iii) fitness challenge: short duration, high-intensity Crossfit™-style workout performed individually with the aim of completing the workout as quickly as possible; (iv) modified games: minor strength and aerobic- based games (e.g., sock wrestling and tag-style games) and small-sided ball games that maximise participation and active learning time (e.g., touch football); and (v) cool down Measurements taken at baseline, 8 months, and 18 months	Muscle/bone strengthening (a) Push-up test, FITNESSGRAM protocol (b) Hand-grip strength (kg)
Muehlbauer et al. (2012) Germany [69]	Total: *n* = 13 Control: *n* = 7 Mean age: 16.9 ± 0.7 Mass (kg): 66.7 ± 7.5 Height (cm): 182.6 ± 6.3 BMI: 20 ± 2.0 Intervention: *n* = 6 Mean age: 16.8 ± 0.8 Mass (kg): 68.8 ± 2.6 Height (cm): 181.8 ± 6.5 BMI: 21.1 ± 1.7	8 weeks, two sessions per week. Exercises; Squats, leg press, calf-raise, hip abduction/adduction, leg extension/ flexion Training volume; 8-week training period with a total of 16 sessions; each session lasted 90 min (10-min warm-up, 70-min resistance training, and 10-min cool down) Training frequency two training sessions a week separated by approximately 48 h. Training intensity 30–40% of the one-repetition maximum. Training intensity was examined for each participant on a fortnightly basis by means of one-repetition maximum tests; if necessary, the training load was adjusted Measurements taken at pre and post-intervention	Muscle/bone strengthening (a) Maximal isometric force, leg press (b) Rate of force development, leg press (c) Counter-movement jump height
Muntaner-mass and Palou (2017) Spain [70]	Total: *n* = 83 Control: *n* = 35 Mean age: 15.8 ± 0.5 Mass (kg): 64.0 ± 10.8 BMI: 21.1 ± 3.0 Intervention: *n* = 45 Mean age: 15.9 ± 0.6 Mass (kg): 64.7 ± 12.0 BMI: 21.4 ± 3.3	5 months, two sessions per week The intervention consisted of a circuit of ten stations, where a high-intensity activity was performed at each one. The authors do not provide a list of the activities at each station to discuss the movements utilised. Due to the large number of movements delivered at a high intensity, this study was categorised as combined activities Measurements taken at pre- and post-intervention	Muscle/bone strengthening (a) Hand-grip strength (kg) left (b) Hand-grip strength (kg) right (c) Standing broad jump (cm)
Weeks and Beck. (2012) Australia [71]	Total: *n* = 46. Control: *n* = 24 Mean age: 13.8 ± 0.4 Mass (kg): 58.6 ± 16.7 Height (m): 1.640 ± 0.086 BMI: 20.5 ± 4.3 Intervention: *n* = 22 Mean age: 13.8 ± 0.4 Mass (kg): 55.0 ± 13.8 Height (m): 1.637 ± 0.098 BMI: 20.3 ± 3.6	8 months, two sessions per week consisting of 10 min Delivered at the beginning of a physical education lessons. Each bout of jumping comprised at least some of the following manoeuvres: jumps, hops, tuck jumps, jump squats, stride jumps, star jumps, lunges, side lunges, and skipping. Each 10-min session consisted of 300 jumps Measurements taken at pre- and post-intervention	Muscle/bone strengthening Vertical jump (cm)
Winwood and Buckley. (2017) New Zealand [72]	Total: *n* = 62 Control: *n* = 23 Mean age: 14.3 ± 0.5 Mass (kg): 63.2 ± 13.2 Height (cm): 174.1 ± 8.7 Intervention (bodyweight and mobility): *n* = 25 Mean age: 14.2 ± 0.4 Mass (kg): 64.4 ± 12.2 Height (cm): 175.2 ± 8.1 Intervention (combined bodyweight, mobility and free weight resistance): *n* = 14 Mean age: 14.3 ± 0.5 Mass (kg): 61.8 ± 13.1 Height (cm): 174.0 ± 9.6	7 weeks, 2–3 sessions The 7-week training intervention involved participants performing 2 body weight/mobility training sessions per week (Table 2), which was in addition to their regular sport training. While session 1 had a focus on improving strength and session 2 on mobility, each session sought to improve fundamental movement skills. The training program required the participants to train for up to 60 min biweekly on non-consecutive days Participants in the combined training group (CBT) performed two additional 60-min RT sessions in the same week but on different days to the BMT sessions. The focus of the training program was to enhance strength and improve fundamental movement patterns using key multi-joint movements Measurements taken at − 1 and + 2	Muscle/bone strengthening Push-up tests (reps) (a) Bodyweight and mobility (b) Bodyweight, mobility, and weight resistance Horizontal jump (m) (c) Bodyweight and mobility (d) Bodyweight, mobility, and weight resistance Medicine ball throw (m) (e) Bodyweight and mobility (f) Bodyweight, mobility, and weight resistance Counter-movement jump (m) (g) Bodyweight and mobility (h) Bodyweight, mobility, and weight resistance

### Pooled Analysis: Muscular Fitness

**Fig. 3:**
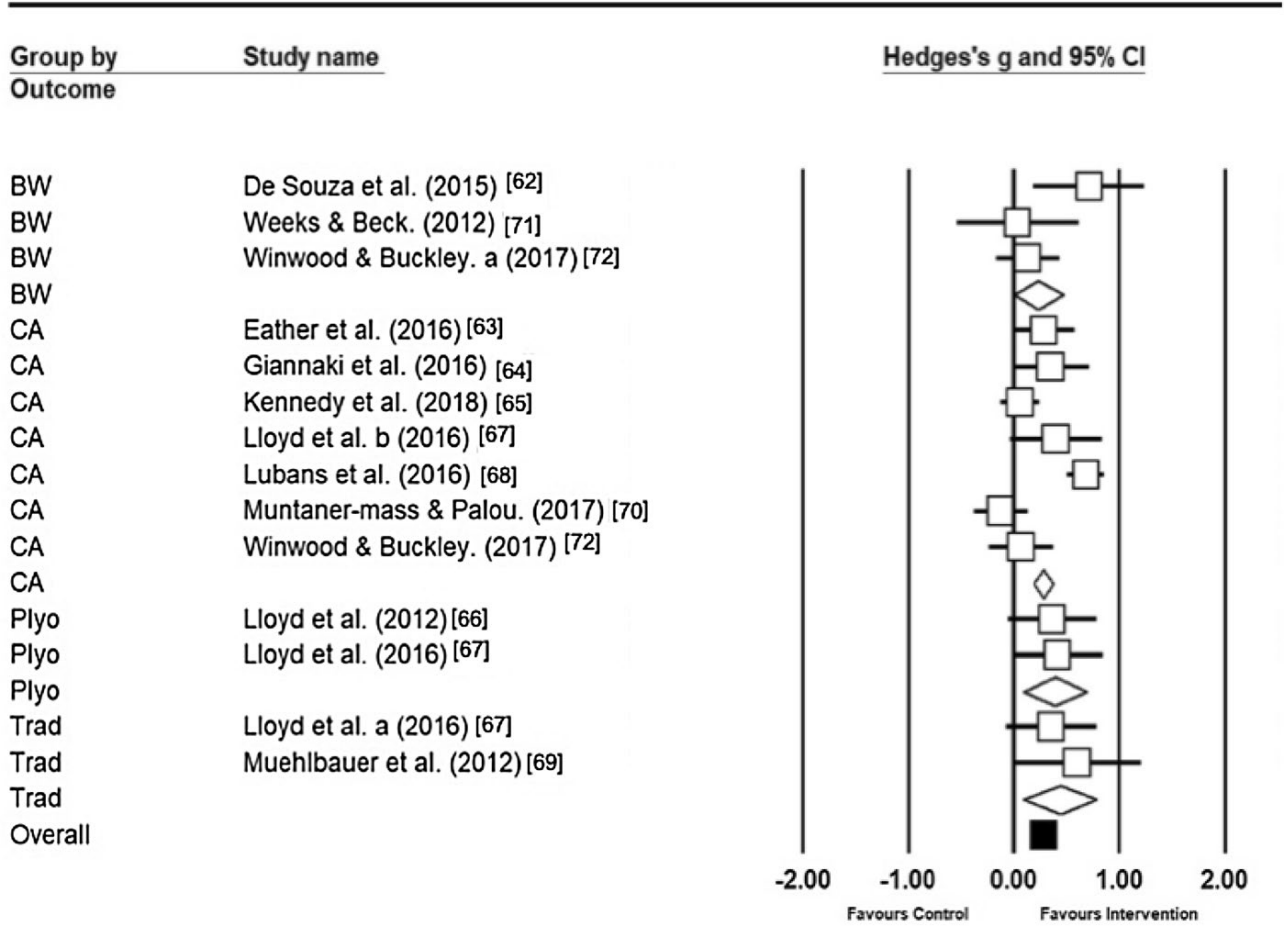
Individual study and pooled results of MF training outcomes. *BW* bodyweight, *Trad* traditional, *Plyo* plyometric, *CA* combined activities. Letters a and b were used to separate studies investigating more than one type of resistance training

Muscular fitness interventions demonstrated an overall small-to-medium effect (*g* = 0.32, CI 0.17, 0.48, *p* < 0.00). Medium-to-high heterogeneity was present amongst the 43 data sets (*I*^2^ = 71.50). The 43 data sets came from 11 studies accounting for different MF outcomes and measures within each intervention and can be seen in Table 2. The overall effect of all interventions investigating MF can be seen in Fig. 3.

### Upper and Lower Limb Activities

**Fig. 4:**
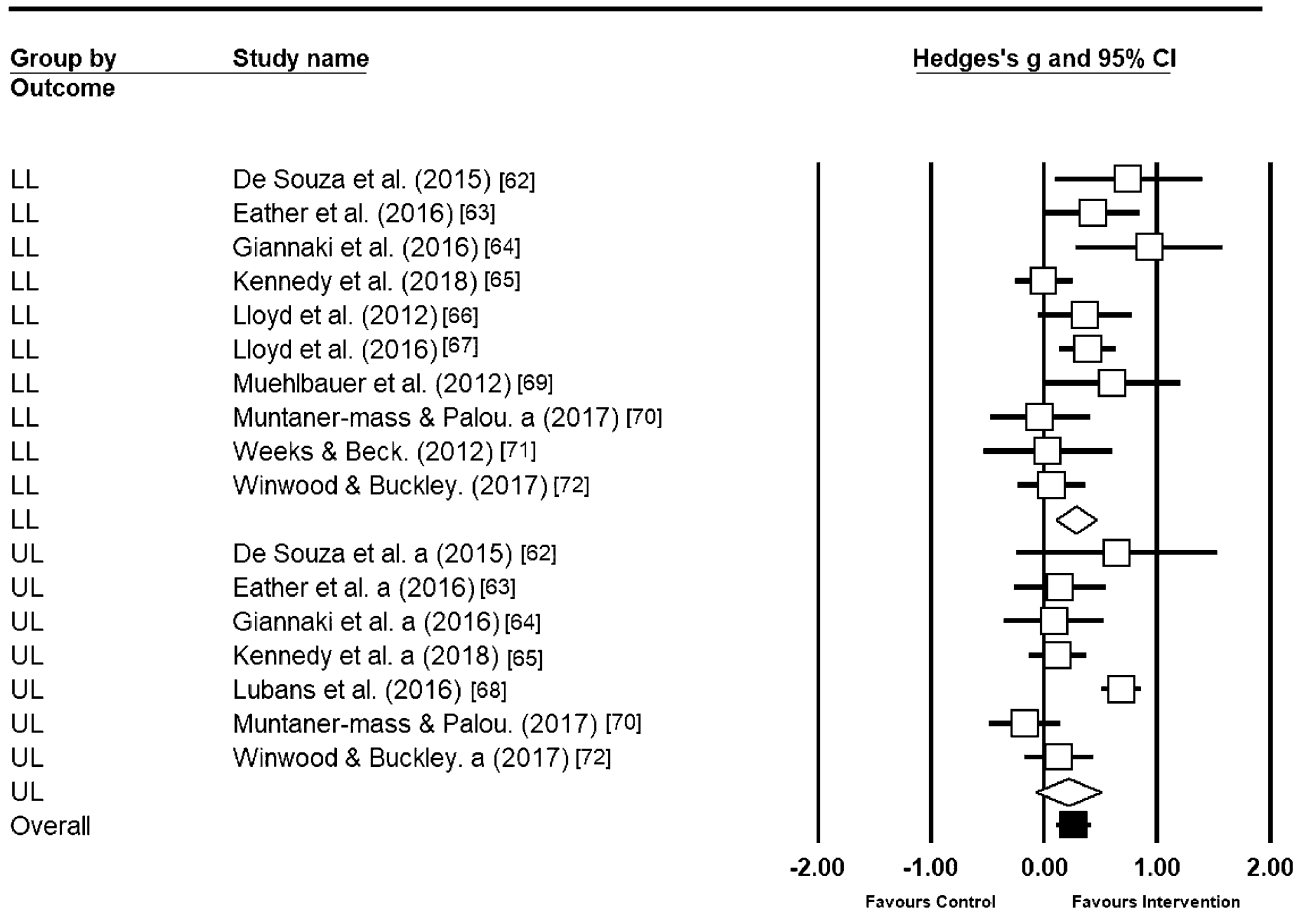
Individual and pooled subgroup analyses of upper limb and lower limb MF outcomes. Studies with more than one outcome of MF are reported separately with the letter a allowing for separation between LL and UL outcomes. *LL* lower limb; *UL* upper limb

Muscular fitness outcomes were separated into those that assessed upper limb (*n* = 14) and lower limb muscle outcomes (*n* = 27). Two data sets measuring core strength were omitted from the analysis as this number was insufficient. Upper limb outcomes presented a small-to-medium effect, with moderate heterogeneity (*g* = 0.28, 95% CI − 0.02, 0.58, *p* = 0.07, *I*^2^ = 83.86). Lower limb outcomes displayed less heterogeneity when compared to upper limb (*I*^2^ = 46.41) and elicited a small-to-medium effect (*g* = 0.28, 95% CI 0.09, 0.68, *p* = 0.03). The corresponding forest plot can be seen in Fig. 4.

### Combined Activities

Combined activities (CA) consisted of those interventions that incorporated multiple methods to enhance MF, such as plyometric, bodyweight, and traditional methods conducted within the same session (*n* = 22). There was a small effect for these interventions (*g* = 0.24, 95% CI − 0.04 to 0.49, *p* = 0.05), which had high heterogeneity (*I*^2^ = 84.86).

### Plyometric Activities

Plyometric forms of training (*n* = 6) resulted in a small-to-moderate effect size (*g* = 0.39, 95% CI 0.09, 0.68, *p* = 0.01). Analysis of heterogeneity demonstrated that plyometric forms of training were homogeneous (*I*^2^ = 0.00).

### Body Weight Activities

Interventions utilising body weight (BW) as the resistance elicited a small effect (*n* = 8, *g *= 0.27, 95% CI − 0.10, 0.65, *p = *0.15). Analysis demonstrated medium heterogeneity (*I*^2^ = 51.53) for all studies utilising BW.

### Traditional Methods

Traditional methods (TM) were deemed to be those methods that utilised free weights and resistance machines [75]. TM indicated a small-to-medium effect (*n* = 7, *g *= 0.43, 95% CI 0.09, 0.78, *p *= 0.01). TM displayed low heterogeneity (*I*^2^ = 0.00) and the greatest effect size in relation to the control groups. The entire breakdown of MF subgroups is presented in Fig. 3.

## Discussion

To date, the literature has primarily focussed on the aerobic MVPA aspect of the PA guidelines, often overlooking MF [76]. Furthermore, adolescent boys are under-represented in the literature relative to girls [77]. This review builds upon the current literature by investigating the MF construct of PA. Our findings demonstrated that MF interventions were effective, which concurs with current literature, suggesting that adolescent boys may be receptive to MF interventions [78]. However, the small-to-moderate findings of this review should be interpreted with caution and considered in light of the high heterogeneity and a lack of specificity regarding the desired MF outcome in the studies. Moreover, the use of the term “strength training” within the literature is often misused, disregarding the independent nature of training adaptations to differing exercise modalities and overlooking the principle of specificity [79]. The concern of inappropriate inference to outcome measure has been recently raised [80] and the findings of this review suggest that there is also a lack of outcome measure specificity for MF and strength training in school-based studies.

The literature suggests that MF interventions lasting 8–12 weeks are most effective in adolescent populations [8, 81, 82]. Seventy-two percent of studies investigating MF interventions met or exceeded this, suggesting that intervention duration may not have been long enough in over a quarter of studies to evoke an efficacious response. It is acknowledged that MF must adhere to underlying physiological characteristics that affect muscular strength to elicit an efficacious response and/ or adaptation [83]. Furthermore, the development of strength is underpinned by a combination of neural and morphological factors that may not be specifically catered for by conducting combined activities that involve high-intensity circuit-based interventions [83]. Adolescence provides an opportunity for neural and architectural adaptations in the development of strength due to increases in anabolic and hormonal concentrations [84]. However, 21 of the 43 data sets investigating MF utilised combined activities and may have overlooked the existing evidence-based methods that educe a more favourable response to the development of MF, such as specific set and repetition schemes combined with appropriate rest periods. However, the practicalities, compliance, and pedagogical considerations associated with designing an MF program have not been explored in the literature, and may explain the lack of clarity on appropriate MF intervention design for a school-based setting. Moreover, the implementation of school-based RT may be impaired by some teachers reporting a lack of expertise/qualification and low confidence in the delivery of PE [85] which may be further exacerbated through the introduction of RT which currently resides outside of traditional PE [86].

Interestingly, plyometric RT demonstrated a statistically significant, homogeneous effect. Plyometric training has been evidenced to benefit peak bone mass in adolescent girls [87], and though evidence in boys is currently lacking, similar responses may be expected. However, only two studies adhered to appropriate plyometric training protocols, supporting the need for further quality research in this method of RT. Plyometric forms of training show promise and may provide a way to enhance muscle and bone strength. However, if such protocols are to be used within schools, appropriate training must be provided to ensure the safety and efficacy of this mode of RT. Moreover, individual variability in biological age, training age, skill, and coordination will dictate prescription of training frequency, intensity, velocity, and volume of plyometric RT [88]. The complexities associated with plyometric RT may explain the lack of research. Thus, consideration to pedagogy and practical application beyond the research in a school-based environment requires further investigation.

A key finding of this analysis was that traditional methods of MF were most effective. These are similar to those commonly practiced in commercial gymnasium environments that adolescents may encounter after leaving school. Thus, exposure to traditional RT may allow for preparation towards the transition into a popular form of PA conducted by adults. Recommendations for loading protocols can expect to see loads of 5–10% added once the individual can comfortably perform 15 repetitions of a given movement with good form [89]. This method of adding load to progress the intensity of the RT may allow for greater perceived autonomy, whilst ensuring load increases are controlled through traditional machines and equipment allowing for smaller incremental increases when compared to bands or bodyweight. Moreover, allowing individuals to regulate the load progressions may enhance the intrinsic appeal [90]. Furthermore, the potential for enhancing physical literacy through neuromuscular adaptation indicative of RT may allow for previously disengaged adolescents to enhance their competence and participate in PA with greater intent and vigour. Adolescents that are overweight or obese may outperform their leaner peers when conducting the traditional forms of RT expressed in an in an absolute manner [51]. This may be due to their increased fat mass being indicative of a higher fat free mass, and thus, obese and overweight individuals may be able to lift or move more weight than leaner adolescents. Collectively, this greater involvement and ability to exercise competently alongside their peer group may allow for the relatedness component of self-determination theory (SDT) to be satisfied. Further research is warranted and should investigate SDT as a psychological construct to inform RT intervention design and content.

Subgroup analyses of muscle group was conducted to explore potential variance in MF outcomes, attributed to growth, maturation, and peak strength velocity occurring approximately 2 years after peak height velocity in adolescent boys [91, 92]. Evidence suggests that children and adolescents have a reduced ability to recruit type 2 muscle fibres, resulting in a lower voluntary muscle strength, speed, and power output [93–95]. Interventions conducted in the school environment may provide variance as to when students reach PHV and in turn, PSV. School-based interventions delivered to a broad range of youth should focus on developing muscle groups that may produce a homogeneous effect across a variety of ages, abilities, environments, and attitudes towards PA. This systematic review and meta-analysis demonstrated that lower limb MF outcomes (*n* = 27) had a homogeneous small-to-medium effect when compared to upper limb outcomes. This is irrespective of the potential for variance in ability, age, and attitude towards PA, and suggests that interventions targeting lower limb may be more effective than interventions designed to target upper limb. However, these results should be interpreted with caution as seven different measures to assess lower limb strength were used throughout the studies. Future research should standardise the use of lower limb strength measurements to assess and contextualise the efficacy of RT and its impact on lower limb development in the school environment. The findings of this review suggest that lower limb strength can be increased in a school-based setting across a broad spectrum of ages, abilities, and body types. Investing more time into the development of lower limb MF may support lowering the high percentage of lower limb injuries currently witnessed in active adolescent males [96], allowing those active individuals to continue PA within and beyond formal education. Furthermore, it has been suggested that the loss of muscle mass associated with the ageing process later in life may result in reductions in PA, with lower limb muscle groups being particularly susceptible to this phenomenon [97]. The findings of this review suggest that school-based interventions may contribute to homogeneous development of lower limb MF in adolescent males and contribute towards mitigating age-related declines through effective and early development of lower limb MF.

Methods of assessing upper limb strength (*n* = 14) were consistent across all seven studies. Press ups and grip strength featured in five and four of the studies, respectively, with one study assessing medicine ball throw. However, grip strength for upper limb assessment may not be the most reflective of those movements conducted during everyday life or as part of an exercise training regime [18, 98]. Recently, back leg and chest dynamometry has been validated in adolescents and may provide a cost effective, mobile, and simple tool to assess overall limb strength [99, 100]. To date, no school-based interventions investigating MF have utilised back leg and chest dynamometry as a measure to assess overall limb strength. Future research should consider the use of back leg and chest dynamometry to provide a measure of overall strength that may be more aligned to everyday life and as a marker of health [18, 99]. Upper limb MF outcomes did not provide a homogeneous outcome despite the consistency in assessment measures. This may be attributed to the variance in ages, both biologically and chronologically having an impact on force generation of the upper limb due to restriction in type 2 muscle fibre utilisation [101]. There may be a pedagogical concern when considering some of the functional shortcomings in adolescent boys, especially when attempting to design intervention and training protocols for this population group [96]. Although data are limited, it is suggested that upper limb RT may account for a larger proportion of injuries in early adolescence [96]. Further research is required to account for the heterogeneity in MF outcomes of the upper limb, and provide practitioners with appropriate, safe, and effective stimulus to enhance MF in adolescent males.

Only two studies objectively measured trunk strength. Trunk strength measures are simple to conduct and may inform the health of the lower back [102]. Although measures of trunk strength are simple to conduct in a field-based setting, researchers may be discouraged by the lengthy familiarisation process [103]. Researchers should explore methods that support a reduced familiarisation period or introduce familiarisation methods before intervention and data collection.

Reporting of the school-based MF interventions is sparse within the literature [104]. Furthermore, the utilisation of behavioural theory and socio-ecological models to underpin the delivery of MF interventions are not widely used. This may be due to recent work, suggesting that these models and constructs may not elicit a favourable outcome in the delivery of PA interventions investigating aerobic MVPA [105–108], resulting in a lack of willing to explore behavioural constructs when designing interventions. The school-based environment is unique in providing a largely mandatory setting to a broad range of youth [109]. Future intervention design may benefit from exploring enhanced, extended, and expanded opportunities (TEO) for youth PA and MF development in conjunction with complex behavioural theories [109] and avoid repeating the shortcomings evidenced in school-based aerobic MVPA intervention design [105–108]. TEO allows for a pragmatic approach to intervention design, expanding on PA opportunity by adding to the current PA opportunities, extending PA by adding additional time to current PA opportunities and, enhancing PA by augmenting existing PA opportunities [109]. Addressing both TEO and motivational psychological constructs may enhance the quality of the PA experience and positively impact intervention outcomes [109]. At an age where adolescent males may be preparing to leave the formal education environment, providing an opportunity to participate in RT may fulfil both a desire [110] and a need to explore a mode of PA that supports lifelong PA [111]. Future research should utilise TEO to allow both teachers and students to become familiar with the prescription of RT through the addition of its use within a school-based setting. This may help dispel some of the myths surrounding implementation (i.e., the need for specialist equipment and RT can damage growth) [112] and cultivate future intervention design.

Although RT in schools is still a developing concept, examples of periodized implementation have been reported when integrating RT [113]. As discussed, the correct implementation of an RT program is reliant upon accurate and appropriate testing to ensure that the practitioner can assign the correct volume and intensity to progress the adolescent [83]. Previously, testing protocols in the school environment have been greeted with trepidation from parents [114]. Traditionally fitness testing has been aerobically, or bodyweight centred, which may negatively impact physical self-concept in overweight and obese adolescents [114–116]. However, the nature of assessing MF can provide a way of overweight and obese adolescents to demonstrate their increased absolute strength when compared to their leaner peers [51]. Highlighting the areas in which adolescents excel physically may support positive relationships with PA, sport, and PE.

In addition to the testing considerations necessary for the implementation of RT interventions, the timing and period of delivery is equally as important [8, 81, 82]. The school environment lends itself well to the development of macrocycles that cover an academic year [113]. Furthermore, the structure of terms within the academic year could provide a way to develop detailed planning lasting between 2–6 weeks in the form of a mesocycle [117]. Consideration to time constraints placed upon the school should be taken into consideration when developing future interventions. Typically, exposure to PA is conducted within PE sessions lasting 45–60 min [118], allowing for a suitable amount of time to conduct effective RT in the school setting [119]. Overall, methods of constructing long-term planning are not only pragmatically appropriate to the school environment, but also widely recognised with RT literature, in both youth and adults [83]. Future research should consider the potential for the academic year to act as a construct for periodisation, whilst adhering to recognised protocols for RT to enhance specific MF adaptations. RT in schools should be approached with an informed appreciation for the nuances involved in program design, delivery, and a clear objective of the MF adaptation required. For delivery success at a larger scale, training must be provided to teachers and school coaches to confidently and effectively deliver RT.

## Strengths of this Review and Meta-analysis

To the authors’ knowledge this review is the first to address the efficacy of school-based PA interventions on MF outcomes in adolescent boys. This systematic review and meta-analysis are novel by way of addressing MF outcomes which are an element of youth PA guidelines. Further strengths were that the process to locate and extract all relevant data was rigorous and utilised an experienced librarian to ensure a comprehensive search strategy. Moreover, the grey literature search ensured that relevant non-peer-reviewed information was not missed.

## Limitations and Recommendations for Future Research

There are limitations to this study that should be considered when interpreting the results. Although this review aimed to provide an international reference based upon the publication of the WHO PA guidelines [1], it should be noted that recommendations for RT were made in the 2008 American PA guidelines [120] and in earlier publications [121]. However, many countries are yet to develop their own PA policy and may utilise the WHO PA guidelines [1] as a global reference to inform their national PA guidelines and policy [54]. Furthermore, continuity of assessment method for MF interventions varied greatly, especially in the lower limb. The way in which training regimes were administered may also impact the outcome within the interventions, it is well understood that the end result of MF is determined by how the intervention is delivered and further research should seek to contextualise this to appropriately inform future practice [83]. Future research should investigate how differing MF delivery impacts the efficacy and outcome of the intervention.

Additionally, qualitative measures should be utilised to address the concerns of adolescent boys reported within the literature, with a third reporting a desire to enhance muscular aesthetics and another third reportedly wanting to become leaner [122, 123]. Furthermore, it has been hypothesised that adolescent boys may be more inclined to participate in MF activities that are deemed more masculine [45]; this may have an impact on habitual PA. To date, the literature investigating the potential effect enhancing MF has on habitual PA has not been appropriately investigated and requires further work. Due to an insufficient amount of studies available reporting MF outcome aim (i.e., muscular endurance and power), analysis of specific adaptation outcomes could not be completed. Future research should be encouraged to provide an outcome measure such as increasing muscular endurance, power, or hypertrophy, so that future inferences and recommendations can be based upon the intervention outcome.

Future research should standardise MF assessment methods for use within adolescent population groups. Accurate measures of MF outcomes should be a documented within the literature to provide reliable measurement tools. Poor reliability may lead to erroneous conclusions about the MF parameter being measured. Studies investigating changes in MF should consider the whole intervention and how conflicting training modalities may impact MF outcomes. Finally, analysis of further moderators such as age (chronological and biological) and method of delivery (i.e., teacher or researcher delivered) was not possible due to insufficient detail contained within the literature. Future research should consider the impact of age and delivery method during interventions and report the methods within the study.

## Conclusions

This systematic review and meta-analysis found a significant small effect for school-based MF interventions in adolescent boys. Efforts should be made to investigate the often overlooked MF element of the PA guidelines which promote and support physical and psychological health in youth. Traditional and plyometric methods of RT demonstrated the greatest effect when compared to other forms of RT, such as body weight movements, and require further research to draw more generalisable conclusions to inform long-term intervention design.

## Electronic supplementary material

Below is the link to the electronic supplementary material.

Supplementary material 1 (DOCX 218 kb)

## Data Availability

After publication, all data necessary to understand and assess the conclusions of the manuscript are available to any reader of Sports Medicine.
