# Supplementary material for: Efficacy of School-Based Interventions for Improving Muscular Fitness Outcomes in Adolescent Boys: A Systematic Review and Meta-analysis
Source: Sports Med. 2019 Nov 15;50(3):543–60. doi: 10.1007/s40279-019-01215-5 (PMC7018678; doi:10.1007/s40279-019-01215-5)
Supplement: Supplementary file 1 — Supplementary material 1 (DOCX 218 kb) [file 40279_2019_1215_MOESM1_ESM.docx]

Efficacy of school-based interventions for improving muscular fitness outcomes in adolescent boys: A systematic review and meta-analysis

Ashley Cox ¹, Stuart J. Fairclough ¹, Maria-Christina Kosteli ¹, & Robert J. Noonan^2^,

¹ Movement Behaviours, Health and Wellbeing Research Group, Department of Sport and Physical Activity, Edge Hill University, United Kingdom.

^2^ Appetite and Obesity Research Group, Department of Psychological Sciences, University of Liverpool

Liverpool, United Kingdom.

Corresponding author
Ashley Cox: Email 23730170@edgehill.ac.uk ; Twitter @AshCox16

Systematic Search

Search 1

Resistance

| Term | PubMed | SPORT Discus | Web of Science | Google Scholar |
| --- | --- | --- | --- | --- |
| Resistance Training | 18,393 | 10,521 | 22,633 | 4,490,000 |
| Resistance Activity | 139,626 | 4,071 | 188,035 | 4,160,000 |
| Resistance Exercise | 26,801 | 11,665 | 30,855 | 2,530,000 |
| Strength | 273,912 | 68,777 | 956,191 | 5,700,000 |
| Muscle Strengthening | 3,109 | 2,075 | 4,060 | 217,000 |
| Bone Strengthening | 1,062 | 23 | 1,884 | 188,000 |
| Strength Training | 31,755 | 31,151 | 31,947 | 4,740,000 |
| Strength Exercise | 29,754 | 30,312 | 29,986 | 2,820,000 |
| Weight Training | 22,917 | 22,838 | 40,256 | 4,390,000 |
| Weight Lifting | 6,325 | 8,972 | 6,247 | 1,190,000 |
| Weight Bearing Exercise | 4,820 | 968 | 2,373 | 638,000 |
| Bodyweight Exercise | 531 | 301 | 420 | 30,700 |
| Bodyweight Training | 410 | 247 | 257 | 30,500 |
| All Fields (OR) | 283,2157 | 84,718 | 967,050 | 336,000 |

Search 2

Institution

| Term | PubMed | SPORT Discus | Web of Science | Google Scholar |
| --- | --- | --- | --- | --- |
| School | 3,458,408 | 192,659 | 489,958 | 4,990,000 |
| High School | 615,001 | 44,598 | 153,514 | 5,100,00 |
| Secondary School | 284,623 | 12,565 | 40,161 | 4,830,000 |
| Free School | 165,474 | 4,635 | 9,007 | 4,040,000 |
| Pupil Referral Unit | 10 | 2  (n/a) | 28 | 33,700 |
| Grammar School | 616 | 247 | 1,707 | 2,070,000 |
| Comprehensive School | 74,913 | 3,877 | 11,136 | 4,150,000 |
| Private School | 29,279 | 2,140 | 10,776 | 2,650,000 |
| State School | 289,910 | 43,269 | 55,211 | 2,820,000 |
|  |  |  |  |  |
| All Fields (OR) | 3,462,607 | 192,864 | 490,753 | 3,850,000 |

Search 3

Age

| Term | PubMed | SPORT Discus | Web of Science | Google Scholar |
| --- | --- | --- | --- | --- |
| Adolescen* | 1,939,179 | 32,573 | 387,033 | 2,970,000 |
| Teen* | 27,758 | 36,867 | 35,812 | 1,220,000 |
| Puberty | 37,120 | 2,021 | 26,936 | 735,000 |
| Pubescent | 1,903,269 | 174 | 1,402 | 90,100 |
| Youth | 1,923,384 | 28,190 | 125,165 | 3,350,000 |
| Children | 2,269,186 | 103,373 | 1,395,651 | 4,380,000 |
|  |  |  |  |  |
|  |  |  |  |  |
|  |  |  |  |  |
| All Fields (OR) | 3,282,399 | 153,670 | 1,679,889 | 2,970,000 |

**Adjust all further searches for date (2010) as per the initiation of the latest PA guidelines from the WHO**

Search 4

1 AND 2

| Term | PubMed | SPORT Discus | Web of Science | Google Scholar |
| --- | --- | --- | --- | --- |
| Search 1 & 3 | 34,607 | 7,369 | 5,774 | 1,770,000 |

Search 5

1 AND 3

| Term | PubMed | SPORT Discus | Web of Science | Google Scholar |
| --- | --- | --- | --- | --- |
| Search 2 & 3 | 62,984 | 30,762 | 18,147 | 1,432,000 |

Search 6

1 AND 2 AND 3

| Term | PubMed | SPORT Discus | Web of Science | Google Scholar |
| --- | --- | --- | --- | --- |
| Search 1 & 3 & 4 | 4,453 | 1,239 | 3,214 | 3,657,000 |

**Adjust for English Language Only**

Final search (7) for *activities that are strength based*

(“Resistance Training” OR “Resistance Activity” OR “Resistance Exercise” OR Strength OR “Muscle Strengthening” OR “Bone Strengthening” OR “Strength Training” OR “Strength Exercise” OR “Weight Training” OR “Weight Lifting” OR “Weight Bearing Exercise” OR “Bodyweight Exercise” OR “Bodyweight Training”) AND (School OR “High School” OR “Secondary School” OR “Free School” OR “Pupil Referral Unit” OR “Grammar School” OR “Comprehensive School” OR “Private School” OR “State School”) AND (Adolescen* OR Teen* OR Puberty OR Pubescent OR Youth OR Children)

| Term | PubMed | SPORT Discus | Web of Science | Google Scholar |
| --- | --- | --- | --- | --- |
| As above | 4,392 | 1,168 | 3,045 | 500* |

*First 500 results from Google Scholar were used as per the guidance of Haddaway et al (2015).

Haddaway, N. R., Collins, A. M., Coughlin, D., & Kirk, S. (2015). The Role of Google Scholar in Evidence Reviews and Its Applicability to Grey Literature Searching. *PLoS ONE*, *10*(9), e0138237. <http://doi.org/10.1371/journal.pone.0138237>

As per the PRISMA guidelines a link has been provided to one search conducted via SPORTDiscus:

<http://edgehill.idm.oclc.org/login?url=http://search.ebscohost.com/login.aspx?direct=true&db=s3h&bquery=(%26quot%3bResistance+Training%26quot%3b+OR+%26quot%3bResistance+Activity%26quot%3b+OR+%26quot%3bResistance+Exercise%26quot%3b+OR+Strength+OR+%26quot%3bMuscle+Strengthening%26quot%3b+OR+%26quot%3bBone+Strengthening%26quot%3b+OR+%26quot%3bStrength+Training%26quot%3b+OR+%26quot%3bStrength+Exercise%26quot%3b+OR+%26quot%3bWeight+Training%26quot%3b+OR+%26quot%3bWeight+Lifting%26quot%3b+OR+%26quot%3bWeight+Bearing+Exercise%26quot%3b+OR+%26quot%3bBodyweight+Exercise%26quot%3b+OR+%26quot%3bBodyweight+Training%26quot%3b)+AND+(School+OR+%26quot%3bHigh+School%26quot%3b+OR+%26quot%3bSecondary+School%26quot%3b+OR+%26quot%3bFree+School%26quot%3b+OR+%26quot%3bPupil+Referral+Unit%26quot%3b+OR+%26quot%3bGrammar+School%26quot%3b+OR+%26quot%3bComprehensive+School%26quot%3b+OR+%26quot%3bPrivate+School%26quot%3b+OR+%26quot%3bState+School%26quot%3b)+AND+(Adolescen*+OR+Teen*+OR+Puberty+OR+Pubescent+OR+Youth+OR+Children)&cli0=DT1&clv0=201001-201812&type=1&site=ehost-live&scope=site>


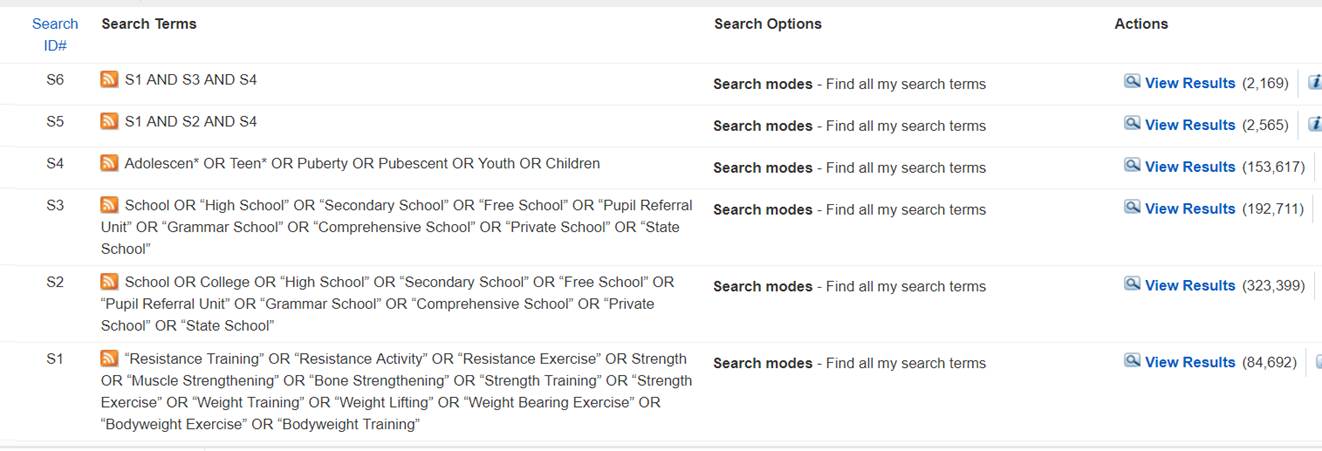


Limiters Applied


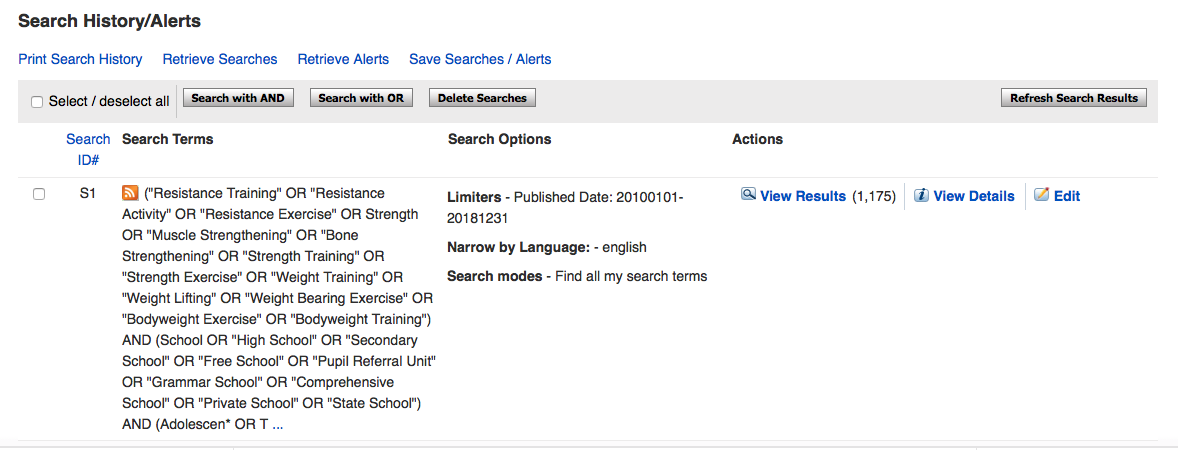


Sense Check Search

Relevant systematic review is sense checked to verify any key literature missing from search. Reference lists from the below reviews were cross-referenced. No additional literature added to covidence.

Collins, H., Fawkner, S., Booth, J. N., & Duncan, A. (2018). The effect of resistance training interventions on weight status in youth: a meta-analysis. https://doi.org/10.1186/s40798-018-0154-z
